# Metabolic Profiling of CSF: Evidence That Early Intervention May Impact on Disease Progression and Outcome in Schizophrenia

**DOI:** 10.1371/journal.pmed.0030327

**Published:** 2006-08-22

**Authors:** Elaine Holmes, Tsz M Tsang, Jeffrey T.-J Huang, F. Markus Leweke, Dagmar Koethe, Christoph W Gerth, Brit M Nolden, Sonja Gross, Daniela Schreiber, Jeremy K Nicholson, Sabine Bahn

**Affiliations:** 1 Biological Chemistry, Biomedical Sciences Division, Faculty of Medicine, Imperial College, London, United Kingdom; 2 Institute of Biotechnology, University of Cambridge, Cambridge, United Kingdom; 3 Department of Psychiatry and Psychotherapy, University of Cologne, Cologne, Germany; University of Queensland, Australia

## Abstract

**Background:**

The identification of schizophrenia biomarkers is a crucial step towards improving current diagnosis, developing new presymptomatic treatments, identifying high-risk individuals and disease subgroups, and assessing the efficacy of preventative interventions at a rate that is not currently possible.

**Methods and Findings:**

^1^H nuclear magnetic resonance spectroscopy in conjunction with computerized pattern recognition analysis were employed to investigate metabolic profiles of a total of 152 cerebrospinal fluid (CSF) samples from drug-naïve or minimally treated patients with first-onset paranoid schizophrenia (referred to as “schizophrenia” in the following text) and healthy controls. Partial least square discriminant analysis showed a highly significant separation of patients with first-onset schizophrenia away from healthy controls. Short-term treatment with antipsychotic medication resulted in a normalization of the disease signature in over half the patients, well before overt clinical improvement. No normalization was observed in patients in which treatment had not been initiated at first presentation, providing the first molecular evidence for the importance of early intervention for psychotic disorders. Furthermore, the alterations identified in drug-naïve patients could be validated in a test sample set achieving a sensitivity and specificity of 82% and 85%, respectively.

**Conclusions:**

Our findings suggest brain-specific alterations in glucoregulatory processes in the CSF of drug-naïve patients with first-onset schizophrenia, implying that these abnormalities are intrinsic to the disease, rather than a side effect of antipsychotic medication. Short-term treatment with atypical antipsychotic medication resulted in a normalization of the CSF disease signature in half the patients well before a clinical improvement would be expected. Furthermore, our results suggest that the initiation of antipsychotic treatment during a first psychotic episode may influence treatment response and/or outcome.

## Abstract 2

Metabolic profiling of the cerebrospinal fluid shows differences between healthy controls and patients with first-onset schizophrenia. Early treatment appears to rapidly normalize the profiles in some of the patients.

## Editors' Summary

**Background.**

Biological markers, or “biomarkers,” are combinations of molecules that are present in certain diseases. Scientists are interested in discovering new biomarkers because they could be useful for diagnosis of those diseases. The presence of such biomarkers might in some cases even precede the development of disease symptoms, which could help in early diagnosis, treatment, and maybe even prevention. Schizophrenia is a disease for which no “objective” biological test exists, and scientists are trying to find biomarkers that would help with diagnosis. The current diagnosis of schizophrenia is based on the symptoms experienced and reported by the patient, in combination with signs observed by a psychiatrist, clinical psychologist, or other clinician.

**Why Was This Study Done?**

This study was done to search for biomarkers for schizophrenia. The researchers studied the metabolic state of patients and healthy volunteers (controls). In other words, they focused on the small molecules present in cells, tissues, or body fluids. The metabolic state reflects what has been encoded by a person's genes and modified by environmental factors. Focusing on the metabolic state makes sense for a disease like schizophrenia, since many different genetic and environmental factors are thought to be responsible for causing it.

**What Did the Researchers Do and Find?**

The researchers studied the metabolic state of 82 patients with schizophrenia and 70 healthy controls by studying the levels of different molecules present in their cerebrospinal fluid (the clear body fluid that surrounds the brain and the spinal cord). Of the patients, 54 had just been diagnosed with schizophrenia (or a similar illness called brief psychotic disorder) and had not yet taken any medications to treat schizophrenia (so-called antipsychotic medication). The remaining patients were undergoing treatment with a range of antipsychotic drugs. The researchers found different levels of certain molecules in the spinal fluid of newly diagnosed patients who had never taken schizophrenia drugs compared with healthy individuals of the same ages. These molecules might therefore turn out to be useful biomarkers for schizophrenia. The differences between patients and controls suggested that the metabolism of several substances—including glucose and acetate—might be altered in the brains of patients with schizophrenia or brief psychotic disorder. The researchers also found that the levels of these molecules in some of the patients with newly diagnosed schizophrenia who were given medication became similar to the levels in the control individuals.

**What Do These Findings Mean?**

These results are encouraging because they suggest that studying “metabolic profiles” might lead to finding a set of biomarkers that could reliably help in early diagnosis of schizophrenia. Such biomarkers might possibly also help in monitoring patients' responses to drug treatment. However, as acknowledged by the study's authors and emphasized by Rima Kaddhurah Daouk in an accompanying Perspective, these early results need to be tested in larger studies and confirmed before their clinical relevance will be known. It will be important for such follow-up studies to involve patients with other psychiatric diseases (not just schizophrenia), to see whether the biomarkers are specific to schizophrenia or whether they indicate a broader range of psychiatric diseases.

**Additional Information.**

Please access these Web sites via the online version of this summary at http://dx.doi.org/10.1371/journal.pmed.0030327.

- National Institutes of Mental Health pages on schizophrenia
- The National Alliance for Research on Schizophrenia and Depression
- The National Alliance for the Mentally Ill
- The Schizophrenia Society of Canada
- Wikipedia page on schizophrenia (note: Wikipedia is an online encyclopedia that anyone can edit)

## Introduction

The current diagnosis of schizophrenia remains subjective, not only because of the complex spectrum of symptoms and their similarities to other mental disorders, but also due to the lack of empirical disease markers. There is a great clinical need for diagnostic tests and more effective drugs for the treatment of severe mental illnesses. An understanding of the molecular characteristics underlying the disease processes of schizophrenia is a fundamental prerequisite to the development of presymptomatic diagnostics and high-value therapeutics.

Biomarkers derived from global expression profiling techniques performed on readily accessible body fluids, such as cerebrospinal fluid (CSF), serum, urine, or saliva, can help identify disease subtypes, aid in predicting and monitoring treatment response and compliance, and identify novel drug targets. These biomarkers could also open up the possibility of developing new early or presymptomatic treatments to improve outcomes or even prevent pathology. Furthermore, the validation of biomarkers that can detect early changes specifically correlated to reversal or progression of mental disorders is crucial for intervention. Used as predictors, these biomarkers could help to identify high-risk individuals and disease subgroups potentially useful as targets for chemo-intervention trials, whilst as surrogate endpoints, biomarkers may be useful for assessing the efficacy and cost effectiveness of preventative interventions at a speed that is not possible when the incidence of manifest mental disorder is used as the endpoint.

^1^H nuclear magnetic resonance (NMR) spectra of biofluids and tissues provide a characteristic “fingerprint” for a range (approximately 20 metabolites can be reliably detected and quantified in CSF) of biologically important endogenous metabolites, such as glucose, lactate and amino acids [1–9]. Quantifiable differences in metabolite patterns in biofluids and tissues can give insight into the underlying molecular mechanisms of disease and can be characterized using automated computer programs that present each metabolite measured in the biofluid spectrum as a coordinate in multidimensional space. Successful application of this technology has achieved identification of biomarkers for a wide range of pathologies, including inborn errors of metabolism, liver and kidney disease, cardiovascular disease, insulin resistance, and neurodegenerative disorders, amongst others [3,4,10–13]. Although a wealth of disease studies have been performed on biofluids such as urine and plasma, relatively few metabolite profiling studies have been performed on CSF for the purpose of disease diagnosis [14–19]. In this study, we undertook an extensive metabolic profiling analysis of CSF samples from control individuals and patients with schizophrenia (note that four of the 54 patients investigated had a diagosis of brief psychotic disorder; i.e., these patients presented with symptoms of paranoid schizophrenia, but had not been ill for a minimum of six months at the time of sample collection, which is required for a DSM IV diagnosis of schizophenia) using ^1^H NMR spectroscopy in combination with computerized pattern recognition analysis. The aim of this investigation was to identify and characterize a possible altered biochemical profile of the CSF in patients with schizophrenia and to determine the metabolic impact of typical and atypical antipsychotic drugs in drug-treated patients with schizophrenia.

## Methods

### Clinical Samples

The Ethical Committee of the Medical Faculty of the University of Cologne (Cologne, Germany) reviewed and approved the protocol of this study and the procedures for sample collection and analysis. All study participants gave their written informed consent. All clinical investigations were conducted according to the principles expressed in the Declaration of Helsinki. CSF samples were collected from drug-naïve patients diagnosed with first episode paranoid schizophrenia (hereafter referred to as schizophrenia) or brief psychotic disorder (*n* = 4) due to duration of illness (DSM-IV 295.30 or 298.8; *n* = 54) and from demographically matched healthy volunteers (*n* = 70) (Table 1). Additionally, samples from patients fulfilling DSM-IV criteria of schizophrenia (DSM-IV 295.30) undergoing treatment with either typical (total *n* = 6: fluphenazine, *n* = 1; haloperidol, *n* = 4; and perazine, *n* = 1) or atypical (total *n* = 22: amisulpride, *n* = 1; clozapine, *n* = 1; olanzapine, *n* = 9; risperidone, *n* = 8; quetiapine, *n* = 2; and ziprasidone *n* = 1) antipsychotic medication were also included. Thus, in addition to the healthy volunteer group, four groups with schizophrenia were examined in this study: two cohorts of drug-naïve patients with first-onset schizophrenia (*n* = 37 and *n* = 17, respectively), one group of patients with schizophrenia who were treated with typical antipsychotic medications, and one group of patients with schizophrenia who were treated with atypical antipsychotic medications.

**Table 1:**
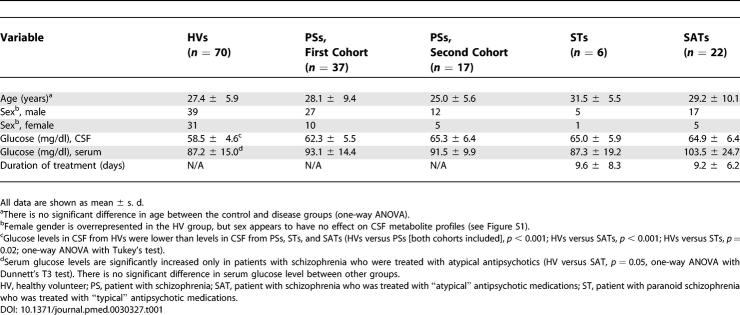
Demographic Details and CSF and Serum Glucose Levels of Patients

Due to an overrepresentation of females in the healthy volunteer group we examined the effect of gender on the metabolite profile, but did not find a gender-specific effect (see Figure S1). We also examined the influence of recent and lifetime cannabis use, determined by urine drug screen and clinical interview, respectively (see Figure S2 and Results).

All samples were collected in a standardized fashion by the same team of experienced clinicians using a nontraumatic lumbar puncture procedure. Trained clinical psychiatrists performed psychiatric assessments. Glucose levels in CSF and serum from healthy participants and patients with schizophrenia were measured immediately after collection using a NOVA BioProfile analyser (Nova Biomedical, Waltham, Massachusetts, United States). CSF samples were divided into aliquots and stored at −80 °C. None of the samples underwent more than two freeze-thaw cycles prior to acquisition of NMR spectra. All experiments were performed under blind and randomized conditions. The volume of CSF samples (150 μl) was made up to a final volume of 500 μl by the addition of D_2_O in preparation for ^1^H NMR analysis.

### ^1^H NMR Spectroscopy of CSF Samples

Standard one-dimensional 600 MHz ^1^H NMR spectra were acquired for all samples using a presaturation pulse sequence to effect suppression of the water resonance (pulse sequence: relaxation delay-90°-*t*_1_-90°-*t*_m_-90°-acquire FID; Bruker Analytische GmbH, Rheinstetten, Germany). In this pulse sequence, a secondary radio frequency irradiation is applied specifically at the water resonance frequency during the relaxation delay of 2 s and the mixing period (*t*_m_ = 100 ms), with *t*_1_ fixed at 3 μs. Typically 256 transients were acquired at 25 °C into 32,000 data points, with a spectral width of 6000 Hz and an acquisition time of 1.36 s per scan. Prior to Fourier transformation, the FIDs were multiplied by an exponential weight function corresponding to a line-broadening factor of 0.3 Hz.

### Data Reduction and Pattern Recognition Procedure

To efficiently evaluate the metabolic variability within and between biofluids derived from patients and controls, spectra were digitally analysed using the software program AMIX (Analysis of MIXtures version 2.5, Bruker Rheinstetten, Germany) and exported into SIMCA-P (version 10.5, Umetrics AB, Umeå, Sweden), in which a range of multivariate statistical analyses were conducted. Initially principal components analysis was applied to the data in order to discern the presence of inherent similarities in spectral profiles. Only one spectrum was excluded from the analysis on the basis of the Hotelling's t-test, which provides a 95% confidence value for a model based on the sample composition. Poor water suppression and high citrate composition were the main cause of sample exclusion. Where the classification of ^1^H NMR spectra was influenced by exogenous contaminants, the spectral regions containing those signals were removed from statistical analysis. In order to confirm the biomarkers differentiating the patients with schizophrenia from matched controls, partial least square discriminant analysis (PLS-DA) was employed.

### Orthogonal Signal Correction of NMR Data

The orthogonal signal correction (OSC) method removes variation in the data matrix between samples that is not correlated with the Y-vector [20]. The resulting dataset was filtered to allow pattern recognition focused on the variation correlated to features of interest within the sample population, which improves the predictivity and separation power of pattern recognition methods [4].

### Other Statistical Methods

Where appropriate, data were subjected to one-way ANOVA using the Statistical Package for Social Scientists (SPSS/PC+; SPSS, Chicago, Illinois, United States). Where the F ratio gave *p* < 0.05, comparisons between individual group means were made by Tukey's test for post-hoc comparisons when the variance was equal between groups. Dunnett's T3 test was used for post-hoc comparisons if variances are not equal. Significance levels were set at *p* = 0.05. Experimental and clinical data were stored in distant research sites, and correlations and subgroup analyses were performed blind.

## Results

### Alterations of CSF Metabolic Profiles in First-Onset, Drug-Naïve, Patients with Schizophrenia

**Figure 1:**
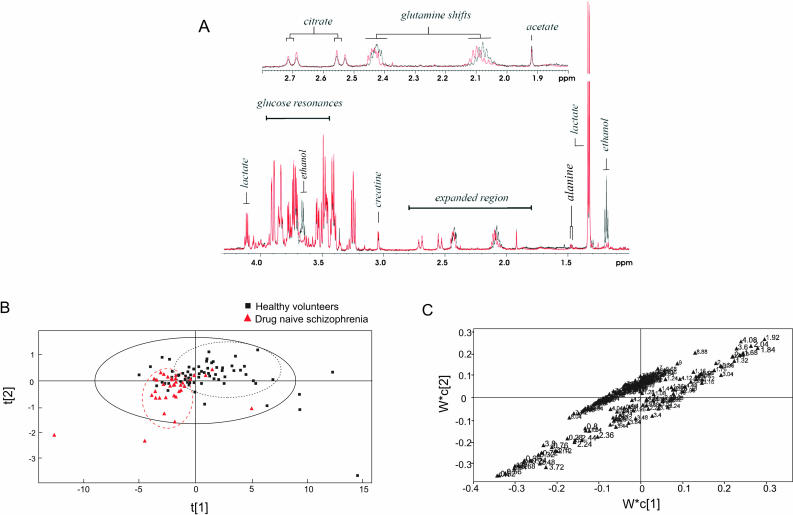
Metabonomic Analysis of CSF Samples from Drug-Naïve Patients with Schizophrenia (A) Partial ^1^H NMR spectrum of a CSF sample from a representative drug-naïve patient with first-onset schizophrenia (red) and a matched control (black) illustrate a characteristic pH-dependent shift in the β-CH_2_ and γ-CH_2_ resonances of glutamine. The prominent signals at ~3.7 and 1.2 ppm correspond to ethanol, a contaminant from skin disinfection prior to lumbar puncture. These signals were removed from statistical analysis. (B) PLS-DA scores plot showing a differentiation of drug-naïve patients with schizophrenia from demographically matched controls as determined by the ^1^H NMR CSF spectra. (C) PLS-DA loadings plot showing major contributing variables towards the separation in the PLS-DA scores plots.

Plots of PLS-DA scores based on ^1^H NMR spectra of CSF samples showed a clear differentiation between healthy volunteers and drug-naïve patients with first-onset schizophrenia (Figure 1). The loading coefficients indicated that glucose, acetate, alanine, and glutamine resonances were predominantly responsible for the separation between classes. Results from ^1^H NMR spectroscopy showed significantly elevated glucose concentrations in CSF samples from drug-naïve patients with first-onset schizophrenia, as compared to the demographically matched control group, with a relative increase in concentration of 6.5% (*p* = 0.04; calculated from a distinct resonance signal at 3.68–3.72 ppm). Direct measurements of CSF glucose levels (performed immediately after sample collection) confirmed a highly significant increase in glucose levels in drug-naïve patients with schizophrenia in the first cohort (6.5% increase, *p* = 0.005; Table 1). Serum glucose levels obtained from the same patients with schizophrenia and from healthy participants showed no difference (*p* = 0.24), suggesting a brain and/or CSF-specific elevation in glucose levels. In contrast, acetate and lactate concentrations were reduced (11.5%, *p* = 0.006; and 17.3%, *p* = 0.05 [t test], respectively) in drug-naïve patients with schizophrenia from the first cohort compared to matched controls. Spectral changes corresponding to glutamine and alanine resulted from a pH-dependent change in the chemical shift of these resonances. CSF samples from untreated patients with schizophrenia was on average 0.1 pH units lower than in the matched control samples (*p* < 0.05, t test), which corresponds to a mean chemical shift change of 0.015 ppm for the β-CH_2_ resonance of glutamine and 0.016 ppm for the alanine CH_3_ signal.

### Effects of Antipsychotic Drug Treatment on CSF Metabolic Profile in Patients with First-Onset Schizophrenia

**Figure 2:**
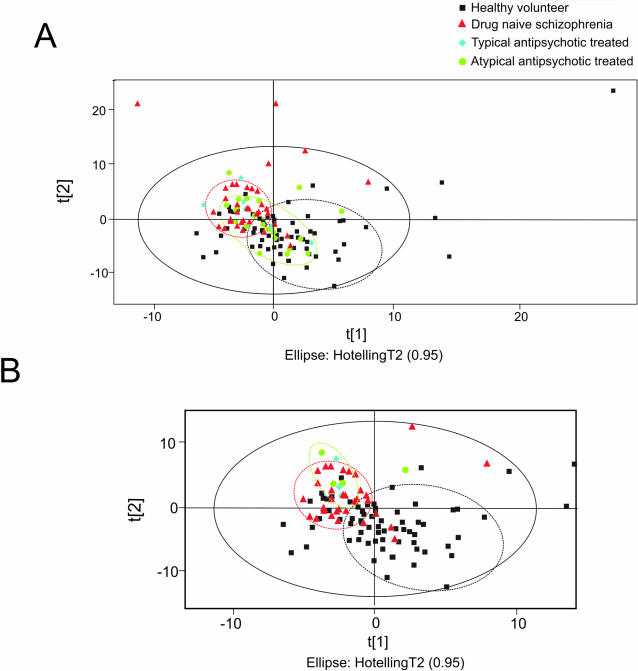
Effects of “Typical” and “Atypical” Medication on CSF Metabolic Profiles in Patients with First-Onset Schizophrenia (A) Spectra from 28 CSF samples from patients with first-onset schizophrenia minimally treated (<9 d, see text for details) with either typical (*n* = 6, blue diamonds) or atypical (*n* = 22, green circles) antipsychotic medications were compared to first onset, drug naïve patients (red triangles) and healthy volunteers (black squares) using PLS-DA. The PLS-DA scores plots show that atypical antipsychotic drug treatment resulted in a shift of approximately 50% of patients with schizophrenia towards the cluster of healthy controls. (B) The same PLS-DA scores plot as (A) except that only minimally treated patients (from both drug groups) with more than one psychotic episode prior to antipsychotic treatment are shown. Note that none of these patients shifted towards the healthy control cluster.

Short-term treatment for an average of about nine days (see Methods and Table 1 for details) with atypical antipsychotic medications resulted in a normalization of the CSF metabolite profile in approximately 50% of patients with schizophrenia (Figure 2A), whereas treatment with one of the typical antipsychotic medications did not show such an effect (Figure 2A). Because the number of patients treated with typical antipsychotic medications is low (*n* = 6), however, no clear conclusions can be drawn from this observation. Interestingly, we observed that patients who suffered several psychotic episodes before drug treatment was initiated (either with typical or atypical antipsychotic medications) did not show a normalization of their CSF disease profiles. Six out of a total of seven patients with more than one psychotic episode before drug treatment cluster closely with the drug-naïve schizophrenia group and, indeed, none of them cluster with the healthy control group (Figure 2B). Moreover, all patients with schizophrenia who exhibited a normalization of the CSF metabolite profile (either with typical or atypical antipsychotic medications) had been commenced on medication during their first psychotic presentation. In statistical terms (recognizing that the numbers are small), our study suggests that if treatment is initiated during a first episode, some of the patients (58% in this study) may recover normal CSF metabolite profiles, whereas if medication is given after a second psychotic episode, normalization may not occur or may be more rare (none of the seven patients showed normalization). Note that experimental and clinical data were stored in distant research sites; all experiments and subsequent data analyses were performed blind to avoid experimental bias.

Due to the prevalent cannabis use amongst patients with schizophrenia and the known effect of cannabis on glucoregulation, we examined the influence of this potential confounding factor in the disease and control groups. None of the control patients had tested positive on urine drug screen and no change in CSF metabolites was observed between healthy volunteers who reported moderate (20–50 times per lifetime) or low/no (<5 times per lifetime) cannabis use (unpublished data). In the drug naïve group with schizophrenia, seven patients (out of a total of 37 in the first cohort) tested positive for cannabis on urine drug screen. Cannabis-positive patients had significantly lower serum glucose levels (9% decrease; *p* = 0.05, t test), but no effect on CSF glucose levels was observed (*p* = 0.20, t test; see Table S1). Three patients who tested positive for cannabis were found to have highly altered CSF metabolite profiles and formed a separate cluster in the PLS-DA plot (away from both healthy controls and patients with schizophrenia) whilst the remaining four cannabis-positive patients clustered with the drug-naïve schizophrenia group (Figure S2).

### Validation of Key Metabolic Alterations in an Independent Test Sample Set

**Figure 3:**
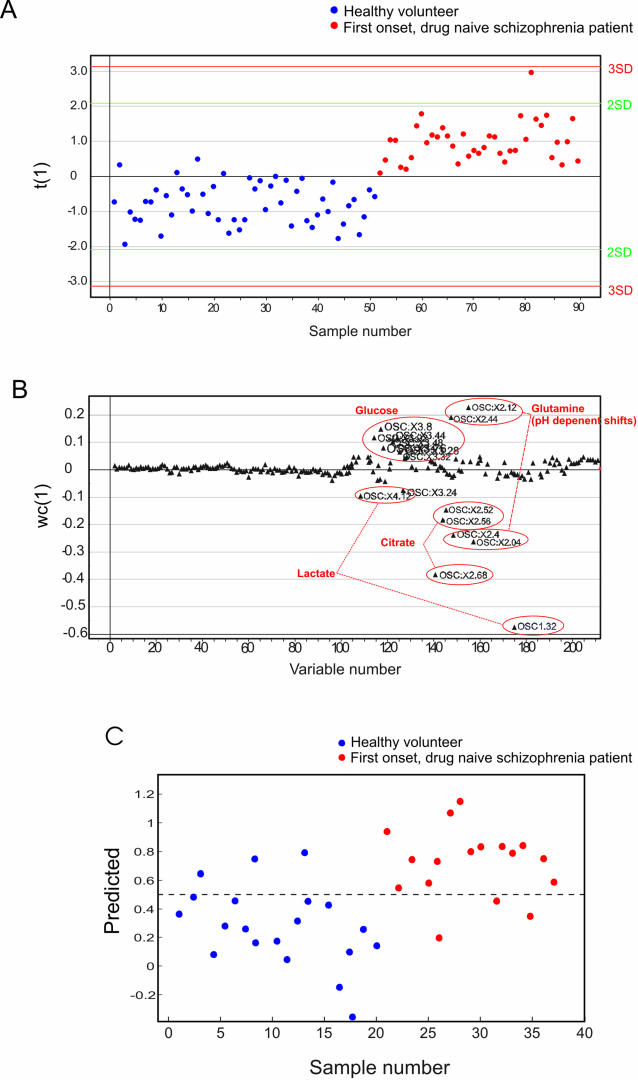
Validation and Prediction of Schizophrenia Group Membership Using a PLS Model A PLS model was constructed using the OSC-filtered data from 37 drug-naïve patients with first-onset schizophrenia from the first cohort (red points) and 50 randomly selected healthy volunteers (blue points) (the “training set”). The scores plot (A) and the loadings plot (B) indicate the key resonances contributing to the separation: lactate, glucose, glutamine, and citrate. This model was then used to predict “group membership” (i.e., schizophrenia or control) in a test set of 17 drug-naïve patients (second cohort) with first-onset schizophrenia and the remaining 20 healthy volunteers which had not been used in the construction of the model. Predictions are made using a Y-predicted scatter plot with an a priori cut-off of 0.5 for class membership (C).

To validate our findings, we reanalyzed samples from the first cohort (70 controls and 37 drug-naïve patients with first-onset schizophrenia CSF samples), alongside a second cohort of 17 additional drug-naïve patients with first-onset schizophrenia. A model was built based on a training set of 50 randomly selected control samples and the 37 samples from drug-naïve patients with first-onset schizophrenia from the first cohort. Both principal components analysis and PLS-DA showed results similar to those in Figure 1 (see also Figure S3). This model was then used to predict class membership in a test set comprising 20 control CSF samples (from the first cohort) and 17 drug-naïve patients with first-onset schizophrenia (from the second cohort, Table 1). OSC was applied to enhance the metabolic differentiation between classes within the model [4]. After OSC, separation of control and drug-naïve with first-onset schizophrenia groups in the PLS scores plots (Figure 3A) was characterized by similar spectral regions as previously identified to contribute to the separation of the classes—i.e., glucose, lactate, shifts in glutamine resonances, and citrate (Figure 3B). The PLS model calculated from OSC-filtered NMR data was then used to predict class membership in the test sample set. The Y-predicted scatter plot assigned samples to either the control or the schizophrenia group using an a priori cut-off of 0.5, and showed the ability of ^1^H-NMR metabonomics analysis to predict class membership of unknown samples with a sensitivity of 82% and a specificity of 85% (Figure 3C).

## Discussion

Analysis of the ^1^H NMR spectra of CSF samples showed a differential distribution of metabolites in samples from healthy volunteers away from drug-naïve patients with first-onset schizophrenia (Figure 1B and 1C). The metabolic profile of CSF was characteristically altered in patients with schizophrenia, and the majority of key metabolites contributing to the separation were replicated in an independent test set (Figure 3). There was some overlap between the first cohort of drug-naïve patients with first-onset schizophrenia and controls in the PLS-DA scores plot derived from the NMR spectra (Figure 1B and 1C). Whilst the drug-naïve patients with first-onset schizophrenia clustered very tightly together, a small number of samples did not show a clear separation in the PLS-DA analysis. This may indicate the existence of schizophrenia subgroups, and it will be of interest to explore whether clinical parameters, such as disease progression, severity, and/or drug response relate to distinct metabolic signatures. Although the sample size of this study is too small to make assumptions about patient subgroups, we note that the four patients who clustered with the control group (Figure 1B) had an exceptionally good outcome or recovered fully from a first episode of psychosis.

Abnormal glucose levels in serum have been linked to antipsychotic drug treatment [21,22], yet our observation of an elevation of CSF glucose concentrations in patients with schizophrenia implies that glucoregulatory alterations may be intrinsic to the schizophrenia syndrome as well as brain-specific, since (1) CSF samples collected from drug-naïve, first-onset patients showed significantly increased glucose, and (2) sera from the same patients showed no such glucose elevation. Elevated CSF glucose has not previously been reported for schizophrenia; however, abnormally high fasting glucose tolerance has been observed in serum from first-onset patients [23]. It is of interest that the prevalence of diabetes type II is substantially increased in patients with schizophrenia (15.8%, compared to 2%–3% in the general population) [24]. Studies also found increased plasma levels of glucose and norepinephrine in patients with schizophrenia [25–27], although increased serum glucose and the high prevalence of type II diabetes in these patients have mainly been attributed to antipsychotic drug treatment [21,27]. Indeed, we found serum glucose levels increased in patients treated with atypical antipsychotic medication (Table 1). It is possible that drug treatment precipitates the onset of diabetes in patients with schizophrenia in the context of a co-predisposition, and that both schizophrenia and diabetes type II share common disease mechanisms. Our recent study investigating global gene, protein, and metabolite alterations in schizophrenia postmortem brain tissue demonstrated significant alterations of pathways relating to glucoregulation and mitochondrial function [37], further supporting the notion of disturbed glucose metabolism in the brains of patients with schizophrenia.

Additionally, a significantly lowered CSF pH aligns very well with our observations in postmortem brain and with alterations observed in energy metabolism at large [37]. Several other studies on postmortem brain also found mitochondrial changes in schizophrenia (e.g., [28,29]). The lowered pH observed in CSF in this study may thus be due to alterations in cellular respiration. Surprisingly, however, although we identified an increase in lactate in postmortem brain tissue, we observed a significant decrease in CSF lactate levels in patients with first-onset schizophrenia. At this stage we are unable to pinpoint which metabolite alterations are contributing to the lowered pH in CSF. A possible explanation could be that the “schizophrenia brain” preferentially utilizes lactate over glucose as an energy substrate. Brain lactate is believed to be predominantly produced by astrocytes [30] and is used as energy substrate in brain, especially by neurons under certain physiological conditions [30]. In fact, significant monocarboxylate utilization by the brain was also reported in different pathological states such as diabetes and prolong starvation [31,32].

Acetate was also significantly reduced in the CSF of drug-naïve patients with first-onset schizophrenia. Since a major portion of acetate in the brain is utilised in fatty acid and lipid synthesis [33], the decreased acetate concentration may suggest a compromised synthesis of myelin-related fatty acids and lipids in the schizophrenia brain. Acetate in the brain is primarily derived from *N*-acetylaspartate (NAA), which is hydrolyzed into L-aspartate and acetate by the enzyme aspartoacylase (ASPA) [34]. NAA is synthesized in neuronal mitochondria and transferred to oligodendrocytes, where ASPA liberates the acetate moiety to be used for myelin lipid synthesis [35]. An in vivo reduction in NAA levels in schizophrenia is a well-established observation [36]. Furthermore, we found ASPA transcripts down-regulated in postmortem brain by microarray and quantitative PCR analysis in schizophrenia postmortem brain (−1.78, *p* = 0.09 by microarray; −1.61, *p* = 0.04 by quantitative PCR; *n* = 15 schizophrenia prefrontal cortex and matched controls [unpublished data]). Together with our findings of a significant decrease of acetate in CSF, this lends further support not only for altered NAA metabolism, but also for oligodendrocyte dysfunction, which we and others previously reported [37,38]. Perturbations in CSF acetate concentrations have also been observed in patients with Creutzfeldt-Jakob disease, although in contrast to the current study, Creutzfeldt-Jakob disease was associated with an increase in acetate concentrations [39].

The specificity of the metabolic signature for schizophrenia found in this study remains to be verified. Disturbed glucose metabolism has also been associated with mood and psychotic disorders [40], although to our knowledge none of these studies measured CSF glucose levels. However, the increased concentrations of glucose together with other metabolic perturbations, such as lower levels of acetate and lactate, and a pH-dependent shift in glutamine resonances, may represent a more specific disease diagnostic for schizophrenia.

The effects of two drug treatment regimens—the use of typical and atypical antipsychotic medication—were evaluated using the same analytical methods. Normalization of the metabolite profiles was observed in patients (*n* = 28) who had been treated with atypical antipsychotic medication for an average of about nine days. Figure 2 illustrates a shift of approximately 50% (11 of 22) of patients on atypical antipsychotic medications towards the cluster of healthy controls within the PLS-DA plot. Although certainly preliminary, these results suggest that atypical medication results in a normalization of the metabonomic disease signature. Only between 40% and 70% (according to different sources) of patients with schizophrenia respond to antipsychotic intervention. Clinical response is generally observed only after weeks or months of treatment. Thus it will be of great interest to examine whether normalization of the metabonomic signature is predictive of clinical drug response. If the normalizing response, which occurs after only a few days of treatment, is confirmed in larger future studies, this could revolutionise schizophrenia treatment and drug discovery approaches. As the sample size of individual antipsychotic drugs in this study was too small to evaluate metabolic effects of each drug, further studies are also required to evaluate their specific effects of individual medications.

A striking finding of this study is the effect of number of psychotic episodes prior to commencing antipsychotic treatment on the CSF metabolite profile in patients with schizophrenia. Again, the numbers were relatively small, but of 21 patients who were commenced on antipsychotic medication during their first psychotic episode, 57% clustered with healthy controls, whereas six out of the seven patients who had several psychotic episodes prior to treatment clustered with the group of drug-naïve patients with first-onset schizophrenia (Figure 2B). These results suggest that the initiation of antipsychotic treatment during a first psychotic episode may influence treatment response and/or indeed outcome. This view is in agreement with the Personal Assessment and Crisis Evaluation (PACE) clinic study [41], the Prevention through Risk Identification, Management and Education (PRIME) study [42] and other ongoing studies that support the notion that early identification and treatment of patients at risk of developing schizophrenia may reduce morbidity and adverse outcomes. Metabonomic approaches of profiling CSF may provide a novel avenue of achieving both early diagnosis and aid in monitoring therapeutic intervention for schizophrenia.

Many patients with schizophrenia are recreational cannabis users, and cannabis has a known effect on glucoregulation; thus we examined this potential confounding factor very closely. We found that recent cannabis use was associated with a significant reduction in serum glucose, but observed no influence on the CSF metabolite profile (Figure S2 and Table S1).

In summary, we suggest that the application of metabolite profiling tools can provide an efficient means for early diagnosis of schizophrenia and provide a practical method for monitoring therapeutic intervention by providing metrics for the normalization of biofluid spectra by multivariate comparison with the relevant control profiles.

## Supporting Information

Alternative Language Abstract S1 Translation of Abstract Provided in Chinese by Author Jeffrey T.-J. Huang (25 KB DOC)

Figure S1 Gender Effect on CSF Metabolic Profile in Drug-Naïve Patients with First-Onset Schizophrenia and Control Participants The PLS-DA model demonstrates that gender did not influence the CSF metabolite profile in either healthy volunteers or the group of drug-naïve patients with schizophrenia.
(217 KB DOC)

Figure S2 CSF Metabolite Profiles of Patients with Schizophrenia Who Tested Positive for Cannabis on Urine Drug Screen (189 KB DOC)

Figure S3 Replication of Metabonomic Analysis on CSF Samples from a “Training Sample Set” Comprising 50 Healthy Volunteers and 37 Drug-Naïve Patients with First-Onset Schizophrenia PLS-DA scores plot (A) and loadings plot (B) show profiles and components discriminating between healthy volunteers (in blue) and drug-naïve patients with schizophrenia from the first cohort (in red), indicating a similar result as reported in Figure 1. These samples were independently reanalysed under an identical condition. Note that the key variables are highly similar to those in Figure 1.
(54 KB DOC)

Table S1 Effect of Cannabis Use on Serum and CSF Glucose Levels in Patients with Schizophrenia (26 KB DOC)

### Accession Numbers

The National Center for Biotechnology Information Unigene (http://www.ncbi.nlm.nih.gov/entrez/query.fcgi?db=unigene) accession number of aspartoacylase (ASPA) is Hs.171142.

## Abbreviations

ASPA: aspartoacylase
CSF: cerebrospinal fluid
NAA: *N*-acetylaspartate
NMR: nuclear magnetic resonance
OSC: orthogonal signal correction
PLS-DA: partial least square discriminant analysis
