# Supplementary material for: Metabolic Profiling of CSF: Evidence That Early Intervention May Impact on Disease Progression and Outcome in Schizophrenia
Source: PLoS Med. 2006 Aug 22;3(8):e327. doi: 10.1371/journal.pmed.0030327 (PMC1551919; doi:10.1371/journal.pmed.0030327)
Supplement: Supplementary file 1 [file pmed.0030327.sd001.doc]

Translation of the abstract into Chinese by author Jeffrey T.-J. Huang

**標題**

腦脊髓液代謝分子之掃描分析：早期治療可能改善精神分裂症的疾病發展及結果

**背景**

精神分裂症生物標記的分析與鑑定是改善目前診斷方式、發展新的發病前治療模式、篩檢高危險個體、細分疾病小群(subtype)、以及快速檢驗藥物有效性的重要一步。

**方法與主要發現**

我們運用質普儀以及電腦化模組辨認方式分析了 82 個來自剛發病、在初期用藥階段或未曾用過精神藥物的精神分裂症病患以及 70個健康志願者的腦脊髓液樣品，運用Partial least square discriminant analysis 我們發現精神分裂症病患以及志願者的腦脊髓液樣品可以以一些代謝分子(如葡萄糖及乳酸)的濃度而加以區分。短期非典型精神藥物治療恢復大約百分之五十病患的腦脊髓液代謝分子濃度，而這個恢復遠早於臨床病症的改善。更重要的是，我們觀察到那些沒有在初次發病即開始藥物治療之病患的腦脊髓液代謝分子濃度並沒有改善，這項發現提供了第一個早期治療精神分裂症的分子證據。我們以第二組測試樣品驗證了前述發現，這個模式的敏感度是82%，專一度是85%。

**結論及重要性**

我們的發現建議精神分裂症病患在發病初期腦脊髓液葡萄糖代謝調節過程發生改變，這些腦部專一的變化並非源自藥物治療的副作用。短期非典型精神藥物治療恢復大約百分之五十病患的腦脊髓液代謝分子濃度，而這個恢復遠早於預期臨床病症的改善。作者建議在第一次發病時即予以藥物治療可能可以改善精神分裂症病患對藥物的反應及療效。
