# Supplementary material for: Metabolic Profiling of CSF: Evidence That Early Intervention May Impact on Disease Progression and Outcome in Schizophrenia
Source: PLoS Med. 2006 Aug 22;3(8):e327. doi: 10.1371/journal.pmed.0030327 (PMC1551919; doi:10.1371/journal.pmed.0030327)
Supplement: Supplementary file 2 [file pmed.0030327.sg001.doc]

**Supplementary Figure 1** PLS-DA model demonstrating that gender did not influence the CSF metabolite profile in either healthy volunteers or the drug naïve schizophrenia group.
