# Supplementary material for: Metabolic Profiling of CSF: Evidence That Early Intervention May Impact on Disease Progression and Outcome in Schizophrenia
Source: PLoS Med. 2006 Aug 22;3(8):e327. doi: 10.1371/journal.pmed.0030327 (PMC1551919; doi:10.1371/journal.pmed.0030327)
Supplement: Supplementary file 3 [file pmed.0030327.sg002.doc]

**A**

**B**

**C**

**Supplementary Figure 2.** CSF metabolite profiles of schizophrenia patients who tested positive for cannabis on urine drug screen.

(A) and (B) PLS-DA scores plots showing profiles and discriminating components of cannabis positive (green and red, respectively) vs. drug naïve, cannabis negative, schizophrenia patients.

(C) Localization of cannabis positive (green triangles) drug naïve schizophrenia patients in the PLS-DA plot in relation to healthy volunteers (black) and drug naïve schizophrenia patients who tested negative for cannabis (red).

Note, that patients 153, 159 and 198 show a highly altered metabolite profile (A) and appear to form a separate cluster (C).
