# Supplementary material for: Metabolic Profiling of CSF: Evidence That Early Intervention May Impact on Disease Progression and Outcome in Schizophrenia
Source: PLoS Med. 2006 Aug 22;3(8):e327. doi: 10.1371/journal.pmed.0030327 (PMC1551919; doi:10.1371/journal.pmed.0030327)
Supplement: Supplementary file 4 [file pmed.0030327.sg003.doc]

A

B

**Supplementary Figure 3.** Replication of metabonomic analysis on CSF samples from a “training sample set” comprising of 50 healthy volunteers and 37 first onset, drug naïve schizophrenia patients.

(A and B) PLS-DA scores and loadings plots show profiles and components discriminating between healthy volunteers (●) and drug naive schizophrenia patients (●), indicating a similar result as reported in Figure 1. These samples were independently re-analyzed under an identical condition. Note that the key variables are highly similar to those in Figure 1.
