# Supplementary material for: Metabolic Profiling of CSF: Evidence That Early Intervention May Impact on Disease Progression and Outcome in Schizophrenia
Source: PLoS Med. 2006 Aug 22;3(8):e327. doi: 10.1371/journal.pmed.0030327 (PMC1551919; doi:10.1371/journal.pmed.0030327)
Supplement: Supplementary file 5 [file pmed.0030327.st001.doc]

**Supplementary Table 1.** Effect of cannabis use on serum and CSF glucose levels in paranoid schizophrenia patients.

|  | Paranoid schizophrenia patients with cannabis “positive” in urine  (n=7) | Paranoid schizophrenia patients with cannabis “negative” in urine  (n=30) |
| --- | --- | --- |
| CSF glucose concentration | 60.3 ± 4.3 | 62.9 ± 5.7 |
| Serum glucose concentration | 86.3 ± 9.0 | 95.1 ± 15.3* |

Data are shown as mean ± S. D.

* p=0.05, t test.
